# Trial Sequential Analysis and Updated Meta-Analysis of Fluvoxamine on Clinical Deterioration in Adult Patients with Symptomatic COVID-19 Infection

**DOI:** 10.3390/ijerph20054088

**Published:** 2023-02-24

**Authors:** Chia-Ling Yu, Andre F. Carvalho, Trevor Thompson, Tzu-Cheng Tsai, Ping-Tao Tseng, Chih-Wei Hsu, Yu-Kang Tu, Szu-Nian Yang, Tien-Wei Hsu, Ta-Chuan Yeh, Chih-Sung Liang

**Affiliations:** 1 Department of Pharmacy, Chang-Gung Memorial Hospital, Linkou 244, Taiwan; 2 IMPACT (Innovation in Mental and Physical Health and Clinical Treatment) Strategic Research Centre, School of Medicine, Barwon Health, Deakin University, Geelong, VIC 3220, Australia; 3 Centre for Chronic Illness and Ageing, University of Greenwich, London SE10 9LS, UK; 4 Prospect Clinic for Otorhinolaryngology & Neurology, Kaohsiung 811, Taiwan; 5 Department of Psychology, College of Medical and Health Science, Asia University, Taichung 413, Taiwan; 6 Institute of Biomedical Sciences, National Sun Yat-sen University, Kaohsiung 804, Taiwan; 7 Department of Psychiatry, Kaohsiung Chang Gung Memorial Hospital, Chang Gung University College of Medicine, Kaohsiung 833, Taiwan; 8 Institute of Epidemiology & Preventive Medicine, College of Public Health, National Taiwan University, Taipei 106, Taiwan; 9 Department of Psychiatry, Beitou Branch, Tri-Service General Hospital, National Defense Medical Centre, Taipei 112, Taiwan; 10 Department of Psychiatry, Armed Forces Taoyuan General Hospital, Taoyuan 325, Taiwan; 11 Graduate Institute of Health and Welfare Policy, National Yang Ming Chiao Tung University, Taipei 112, Taiwan; 12 Department of Psychiatry, Kaohsiung Veterans General Hospital, Kaohsiung 813, Taiwan; 13 Department of Psychiatry, Tri-Service General Hospital, National Defense Medical Centre, Taipei 114, Taiwan; 14 Department of Psychiatry, National Defense Medical Centre, Taipei 114, Taiwan

**Keywords:** fluvoxamine, COVID-19, meta-analysis, trial sequential analysis, deterioration

## Abstract

Preliminary meta-analyses suggested that fluvoxamine was effective in treating COVID-19 infection. However, the reliability of this evidence has not yet been examined. MEDLINE, CENTRAL, EMBASE, PsycINFO, and ClinicalTrials.gov were searched to identify any randomized controlled trials (RCTs) from the inception of the databases to 5 February 2023. We used trial sequential analysis (TSA) to examine the reliability of the current existing evidence on the benefits of fluvoxamine on COVID-19 infection. The primary outcome was clinical deterioration, as defined in the original study (reported as odds ratio (OR), with 95% confidence intervals), and the secondary outcome was hospitalization. In the TSA, we used the relative risk reduction thresholds of 10, 20, and 30%. The updated meta-analysis of the five RCTs showed that fluvoxamine was not associated with lower odds of clinical deterioration when compared with a placebo (OR: 0.81; 0.59–1.11). The effect of fluvoxamine lay within the futility boundary (i.e., lack of effect) when using a 30% relative risk reduction threshold. The effect estimates lay between the superiority and futility boundary using the 10% and 20% threshold, and the required size of information was not reached for these two thresholds. The effect of fluvoxamine on the odds of hospitalization was not statistically significant (0.76; 0.56–1.03). In conclusion, there is no reliable evidence that fluvoxamine, when compared to a placebo, reduces the relative risk of clinical deterioration among adult patients with COVID-19 infection by 30%, and a relative risk reduction of 20% or 10% is still uncertain. The role of fluvoxamine as a COVID-19 treatment cannot be justified.

## 1. Introduction

The 2019 coronavirus disease (COVID-19) is caused by severe, acute respiratory syndrome coronavirus 2 (SARS-CoV-2) infection and can result in serious illness leading to hospitalization, intensive care unit admission, and death [1]. A survey reported that the global all-age rate of excess mortality due to the COVID-19 pandemic was 120.3 deaths (113.1–129.3) per 100,000 population [2]. The excess mortality rate exceeded 300 deaths per 100,000 in 21 countries. However, the data on mortality might still be extremely underestimated [2]. Fluvoxamine, a selective serotonin reuptake inhibitor, is considered to have several potential mechanisms for the treatment of COVID-19 infection, especially the potent activity on the sigma-1 receptor [3,4]. The modulation of the sigma-1 receptor can regulate cytokine production through its interaction with the endoplasmic reticulum stress sensor inositol-requiring enzyme 1α and has been shown to decrease inflammatory damage and shock in murine sepsis models [3,4]. Other mechanisms include decreased platelet aggregation, attenuation of mast cell degranulation, interference with endolysosomal viral trafficking, and elevated melatonin levels, which might have an antiviral effect or modulate the cytokine storm in severe COVID-19 infection [5]. Furthermore, fluvoxamine has the advantages of high accessibility and low cost, especially in the era before the development of vaccines and antivirus agents. Therefore, few randomized controlled trials (RCTs) have examined the benefits of fluvoxamine in patients with COVID-19 infection and reported a positive finding [6,7,8]. Lenze et al. first reported on randomized placebo-controlled trials of fluvoxamine for COVID-19, STOP COVID-19 [7]. A total of 152 participants were recruited (80 received fluvoxamine 300 mg per day, and 72 took a placebo). No participants in the fluvoxamine group had clinical deterioration, but six did in the placebo group. This pilot study further encouraged researchers to investigate the anti-COVID effect of fluvoxamine. Reis et al. then reported a larger RCT conducted in Brazil, TOGETHER, with 1497 patients with COVID-19 infection. The TOGETHER trial reported a lower relative risk (RR) of emergency department visits or hospitalization in the fluvoxamine group when compared to the placebo group (RR: 0.68, 95% confidence intervals: 0.52–0.88).

Two previous meta-analyses included the same three RCTs (STOP COVID 1 trial, STOP COVID 2 trial, and TOGETHER trial) and suggested that fluvoxamine was associated with a reduction in risk of clinical deterioration and hospitalization compared with the placebo [9,10]. However, statistically significant meta-analyses that have a few trials or participants have poor credibility, and the intervention effects may be spuriously over- (type I error) or underestimated (type II error) [11]. The possibility of type I errors in the positive findings from the two preliminary meta-analyses of fluvoxamine is uncertain [9,10]. Moreover, to date, no studies have examined whether the required sample size has been reached to validate the positive effect of fluvoxamine on COVID-19.

Trial sequential analysis (TSA) is a methodology used in systematic reviews and meta-analyses to control for type I and II errors. TSA can model the changing precision in the estimates of the effects as the trials are reported and the likely effect of future trial results on the existing body of data [12]. Additionally, the futility analysis of TSA is analogous to the termination of an RCT when interim analysis indicates that the collection of further data is highly unlikely to alter the interim results [12]. Therefore, TSA can be used to determine whether the body of evidence is sufficiently large and consistent and whether the assumed effect is considered unachievable. Besides, TSA could assess whether the required sample size has been reached. 

The aim of the current study was to examine the reliability of the existing evidence regarding the effect of fluvoxamine on clinical deterioration among adult patients with symptomatic COVID-19 infection using TSA. The PICOS (population, intervention, comparison, outcome, study) settings of the current meta-analysis were P: adults patients with symptomatic COVID-19 infection; I: fluvoxamine; C: placebo; O: clinical deterioration (hospitalization, emergency department visit, or death); S: RCTs. Currently, the availability of vaccines and treatments for COVID-19 is increasing; nevertheless, several trials that have been identified in the registry to test the benefit of fluvoxamine for COVID-19 are still ongoing. Our study findings could inform researchers on the place of fluvoxamine in the priority of treatment options for COVID-19 infection.

## 2. Materials and Methods

### 2.1. Search Strategy and Selection Criteria

The protocol of the current systematic review and meta-analysis was registered a priori in OSF (10.17605/OSF.IO/ZMTS8) and conducted according to the Preferred Reporting Items for Systematic Reviews and Meta-analyses (PRISMA) 2020 statement (Supplementary S1) [13]. MEDLINE, Cochrane Central Register of Controlled Trials (CENTRAL), EMBASE, PsycINFO, and Clinical trials.gov were systematically searched without language restrictions to identify RCTs that examined the benefits of fluvoxamine for adult patients with COVID-19 from the inception of the database to 10 September 2022, and the search was reconducted on 5 February 2023. The specific search terms were adapted to each database. In addition, we manually searched the bibliographies of selected studies and reference lists of review articles meeting the inclusion criteria to locate additional relevant studies.

We excluded case reports, case series, and observational studies (e.g., case-control or cohort studies). We also excluded RCTs involving children. Screening and selection of studies were performed independently by four authors (CLY, AFC, TT, ND TCT), with each study assessed by a minimum of two authors. Disagreements were resolved by consulting with the corresponding author. 

### 2.2. Data Extraction and Assessment of Bias

We extracted data on study characteristics, patient populations, interventions, and reported outcomes from included studies or published meta-analyses. The main outcome of the RCTs addressing COVID-19 could be diverse. For example, the TOGETHER defined clinical deterioration as a collection of retentions in a COVID-19 emergency setting > 6 h or transfer to a tertiary hospital because of COVID-19, while the COVID-Out defined clinical deterioration as a collection of emergency department visits, hospitalization, or death (Table 1). Therefore, we defined our primary outcome (clinical deterioration) as hospitalization, emergency department visit, or death. This definition could satisfy most of the included studies. The secondary outcome was hospitalization. Two independent reviewers (PTT, CWH) assessed each study for bias using the Cochrane risk of bias tool for the RCTs [14]. Disagreements have been resolved by consulting with the corresponding author.

### 2.3. Data Analysis

We performed random-effects meta-analyses with restricted maximum likelihood methods to calculate the odds ratio (OR) as the effect measured and the corresponding 95% confidence interval (CI). We assessed whether there was heterogeneity between the results of individual RCTs using the I2 statistics (I2 > 50% indicating heterogeneity). However, I2 has low statistical power when using a small number of studies, and its confidence intervals can be large [15]. Therefore, we estimated the degree of uncertainty associated with the I2 by calculating the 95% CI for I2, which helped clarify the extent of heterogeneity. In addition, we also employed Cochran’s Q test to assess heterogeneity. Publication bias was assessed with a funnel plot and Egger’s test for the primary outcome. 

A subgroup meta-analysis was performed when at least three sets of data were available. We planned to conduct a subgroup analysis for the nonvaccine era versus the vaccine era. We conducted a sensitivity test to exclude studies that could only provide data that partially met our definition of primary outcome. We also conducted a “leave-one-out” meta-analysis to assess how each individual study affects the overall estimate of the rest of the studies. These analyses were carried out using STATA version 16.0 (StataCorp LLC, College Station, TX, USA) and R version 4.1.1 (www.r-project.org, accessed on 5 February 2023). All statistical tests were two-tailed, and *p* < 0.05 was regarded as significant. References were managed using Endnote version X9 (Clarivate Analytics, Philadelphia, PA, USA).

Subsequently, we performed TSA for the primary outcome using Trial Sequential Analysis software (0.9.5.10 Beta version) (www.ctu.dk/tsa, accessed on 5 February 2023), which performs a cumulative meta-analysis by maintaining the overall risk of type I error at 5%. TSA reports an estimation of information size, which is an estimate of the optimum sample size for statistical inference from a meta-analysis, while considering the heterogeneity of the included studies. We used the sample size for the required information size. TSA also provides thresholds for statistical significance (trial sequential monitoring boundaries) and futility boundary (i.e., an effect is not statistically significant despite an optimum sample size), taking into account multiple statistical tests. The risk reduction thresholds of 10%, 20%, and 30% were used for the primary outcome. 

## 3. Results

### 3.1. Study Characteristics and Quality

**Figure 1:**
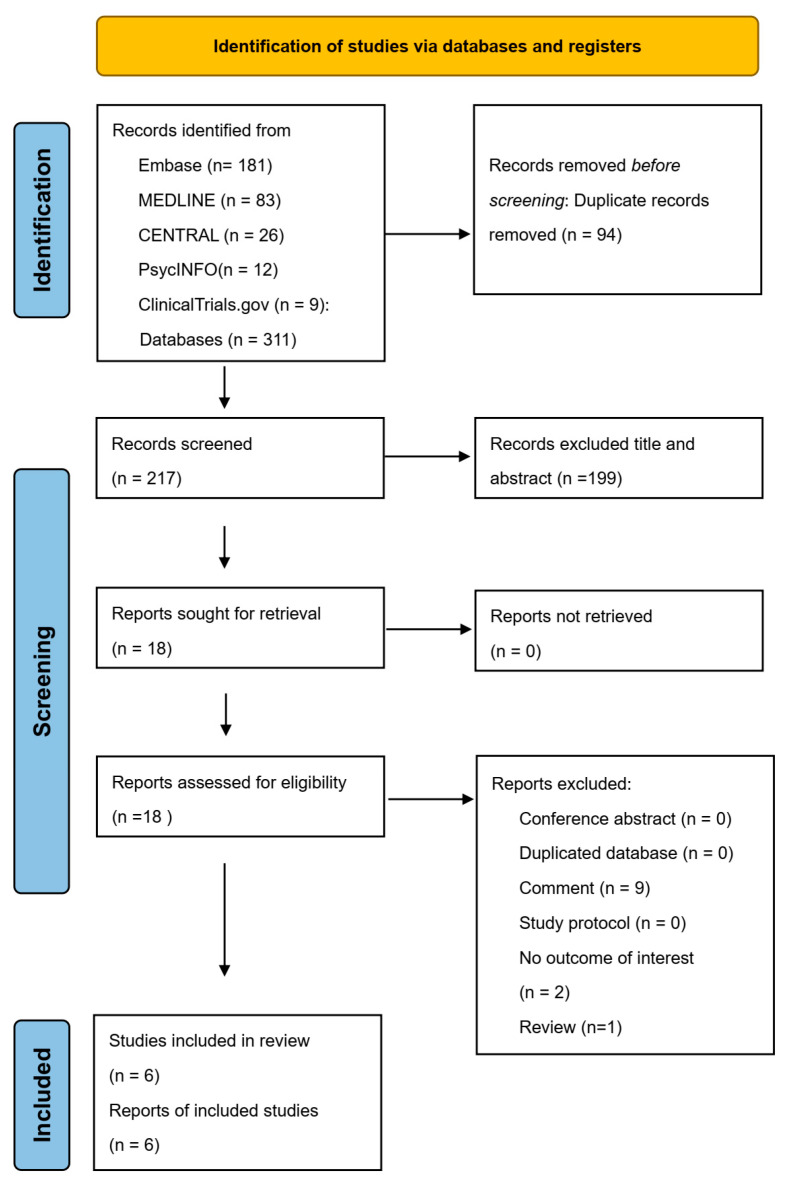
PRISMA 2020 flow diagram.

**Figure 2:**
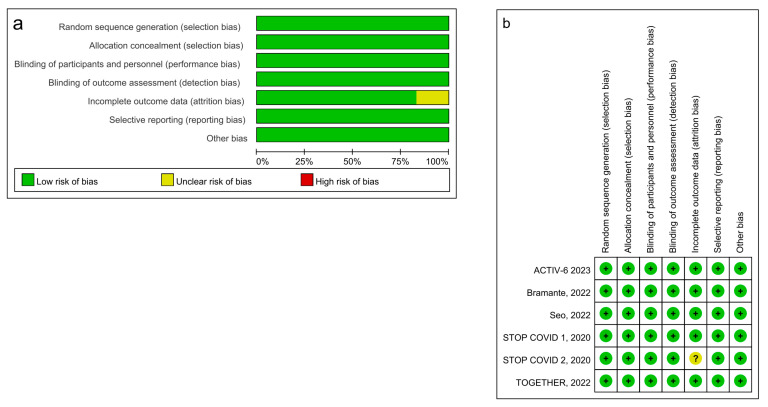
(**a**). Risk of bias graph across the included studies. (**b**). Summary of risk of bias of the included studies ([6,7,8,16,17,18]).

After searching the databases and excluding the duplicate records, we identified 217 potential articles. Finally, six placebo-controlled RCTs with 4178 participants were included in the current meta-analysis (Table 1) [6,7,8,16,17,18]. The flowchart of our search strategy is presented in Figure 1. The complete search strategies (Supplementary S2) and reasons for exclusion (Supplementary S3) [19,20,21,22,23,24,25,26,27,28,29,30] are shown in the online supplement. The six RCTs included 2118 participants in the fluvoxamine group (median age ranged from 46 to 54 years; 31–70% female) and 2060 in the control group (median age ranged from 43 to 52 years; 58–74% female). None of the included studies had a high risk of bias. All six studies had low ROB for randomization, allocation concealment, participant and personnel blinding, outcome assessment blinding, selective reporting, and other biases. Five studies had low ROB, and the other one had unclear ROB for incomplete outcome data. (Figure 2a,b).

**Table 1:** Demographic data of the included studies.

Study; Country	Design	Participants	Intervention	Age (Median, IQR); Female %	Sample Size	Primary Outcome
STOP COVID 1 [7]; United States	Placebo-controlled RCT	Unvaccinated outpatients ≥ 18 years, ≤7-day symptoms	Fluvoxamine 100 mg three time daily	46 (35–58); 70%	80	Clinical deterioration within 15 days: hospitalization or ventilator use due to dyspnea or hypoxia
			Placebo	45 (36–54); 74%	72	
STOP COVID 2 [6]; United States, Canada	Placebo-controlled RCT	Unvaccinated outpatients ≥30 years, ≤6-day symptoms	Fluvoxamine 100 mg two time daily	48 (34–62); 62%	272	Clinical deterioration within 15 days: Presence of dyspnea and/or hospitalization for shortness of breath or pneumonia, decrease in O2 saturation (<92% on room air) and/or supplemental oxygen requirement
			Placebo	48 (35–61); 62%	275	
TOGETHER [8]; Brazil	Placebo-controlled RCT	Unvaccinated outpatients ≥ 18 years, ≤7-day symptoms	Fluvoxamine 100 mg two time daily	50 (39–56); 55%	741	Composite outcome within 28 days: retention in a COVID-19 emergency setting > 6 h, or transfer to a tertiary hospital because of COVID-19
			Placebo	49 (38–56); 60%	756	
Seo 2022 [17]; Korea	Placebo-controlled RCT	Unvaccinated inpatients ≥ 18 years, ≤7-day symptoms	Fluvoxamine 100 mg two time daily	54 (44–60); 31%	26	Clinical deterioration within 15 days: WHO clinical progression scale 4 or greater (hospitalization)
			Placebo	52 (42–59); 60%	26	
COVID-Out [16]; United States	Placebo-controlled RCT	Vaccinated and unvaccinated outpatients, 30–85 years, within 3 days infection	Fluvoxamine 50 mg two time daily	46 (38–53); 51%	329	Clinical deterioration within 14 days: emergency department visit, hospitalization, or death.
			Placebo	43 (37–53); 58%	324	
ACTIV-6 [18] United States	Placebo-controlled RCT	Vaccinated and unvaccinated outpatients, older than 30 years, within 10 days infection	Fluvoxamine 50 mg two time daily	47 (37–57); 57%	670	Clinical deterioration within 28 days: hospitalization, urgent care visit, ED visit, or death
			Placebo	48 (39–58); 57%	607	

RCT: randomized controlled trial, IQR: interquartile range.

### 3.2. Primary and Secondary Outcomes

**Figure 3:**
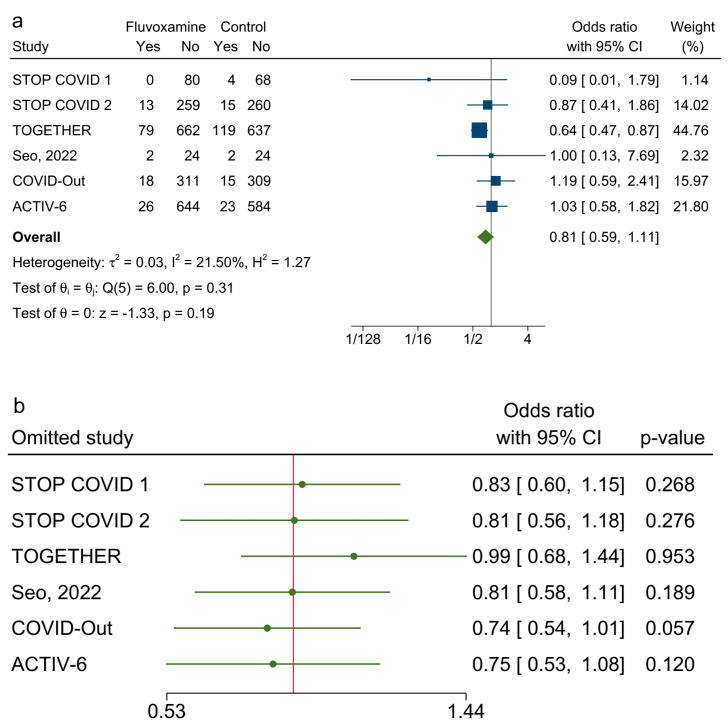
(**a**). Forest plot of random-effects meta-analysis of clinical deterioration. ([6,7,8,16,17,18]) (**b**). The leave-one-out test for the primary outcome. ([6,7,8,16,17,18]).

**Figure 4:**
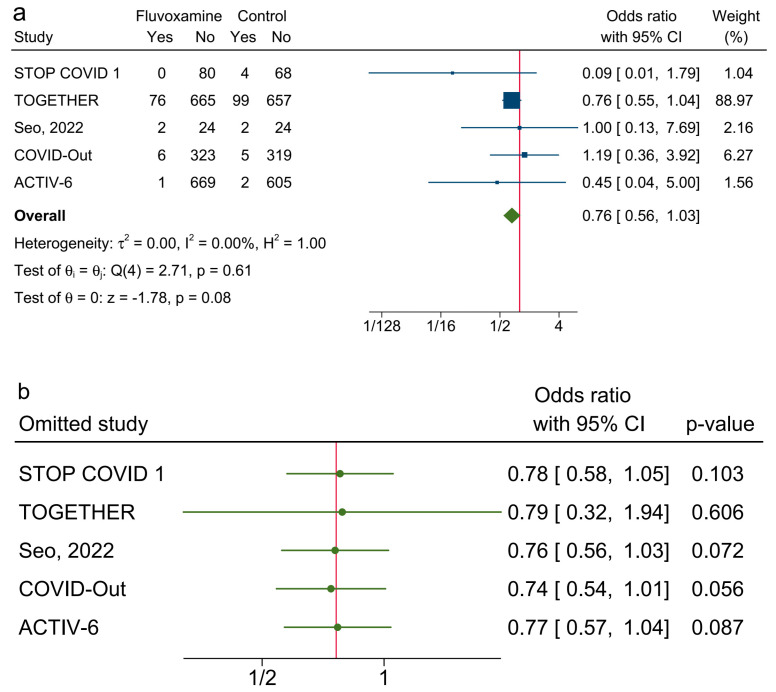
(**a**). Forest plot of random-effects meta-analysis of hospitalization. ([7,8,16,17,18]) (**b**). The leave-one-out test for the secondary outcome. ([7,8,16,17,18]).

Figure 3a shows that fluvoxamine was not associated with reduced odds of clinical deterioration among adult patients with symptomatic COVID-19 infection compared with a placebo (OR: 0.81; 0.59–1.11). The weight was 44.76% for the TOGETHER trial. The point estimate I2 = 21.5%, and the corresponding 95% CI extends from 0.0% to 96.7%. In Cochran’s Q test, the Q value = 6.0, and the *p* value = 0.31. The leave-one-out tests revealed that the pooled ORs were not significant when omitting any of the six studies (Figure 3b). For the secondary outcome, fluvoxamine was not associated with reduced odds of hospitalization (Figure 4a; OR: 0.76; 0.56–1.03). The point estimate I2 = 0% and the 95% CI for I2 = 0% extends from 0.0% to 93.8%. In Cochran’s Q test, Q = 2.7 and *p* = 0.61. The leave-one-out sensitivity test showed that the effect of fluvoxamine was not significant when excluding any of the included studies (Figure 4b). 

### 3.3. Trial Sequential Analysis for the Primary Outcome

**Figure 5:**
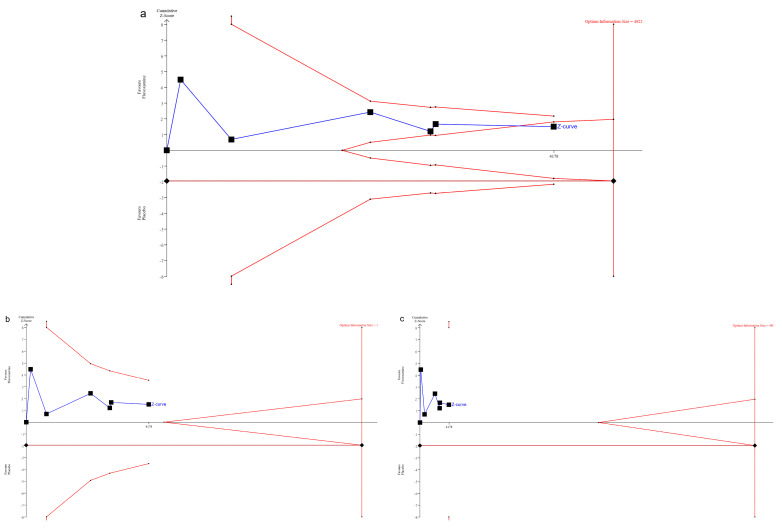
(**a**). Trial sequential analysis of fluvoxamine on the primary outcome using 30% relative risk reduction threshold. (**b**). Using the 20% relative risk reduction threshold. (**c**). Using the 10% relative risk reduction threshold.

Figure 5a shows the results of the TSA using a 30% relative risk reduction threshold. After three RCTs, the z-curve crosses the futility boundary. This indicates that the available evidence is sufficient to suggest that fluvoxamine, when compared with a placebo, did not reduce the relative risk of clinical deterioration by 30% among patients with symptomatic COVID-19 infection. Figure 5b shows that when using a 20% relative risk reduction threshold, the z-curve did not cross the significance boundaries, the futility boundaries, and the line for the required sample size. This indicates that the available evidence is insufficient to support the benefit of fluvoxamine, when compared with a placebo, in reducing the relative risk of clinical deterioration by 20% among patients with symptomatic COVID-19 infection. Figure 5c shows that when using a 10% relative risk reduction threshold, the z-curve did not cross the significance boundaries, the futility boundaries, and the line for the required sample size. This indicates that the available evidence is insufficient to support the benefit of fluvoxamine, when compared to a placebo, in reducing the relative risk of clinical deterioration by 10% among patients with symptomatic COVID-19 infection.

The z-curve is a measure of treatment effect, and the significant boundaries are the thresholds for statistical significance that are adjusted for the heterogeneity of the trial results and multiple statistical testing. A treatment effect outside the significance boundary indicates that there is reliable evidence of a treatment effect, and a treatment effect within the futility boundary indicates that there is reliable evidence of no treatment effect. The required sample size indicates the calculated optimum sample size for statistical inference. A z-curve across the line of the required sample size indicates that the number of participants in the current meta-analysis is sufficient to reach a conclusion.

### 3.4. Publication Bias, Subgroup Analysis, and Sensitivity Test

**Figure 6:**
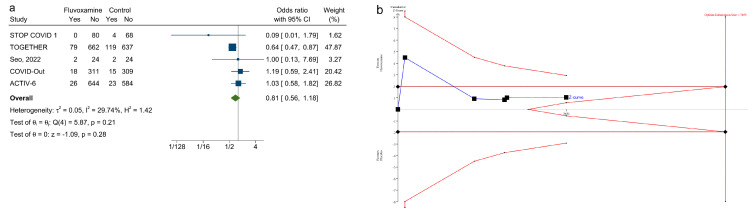
(**a**). Forest plot of random effects meta-analysis, excluding the STOP COVID 2 study in clinical deterioration. ([7,8,16,17,18]) (**b**). Trial sequential analysis of the sensitivity test excluding STOP COVID 2 ([6]) on the primary outcome using a 30% relative risk reduction threshold.

In the sensitivity test, when excluding STOP COVID-2 [6] (from which we could not separate the data for hospitalization, emergency department visit, or death from the data of desaturation and dyspnea), fluvoxamine remained unassociated with reduced odds of clinical deterioration (Figure 6a OR:0.81; 0.56–1.18, I2 = 29.7%, Q value = 5.87, *p* = 0.21). In the TSA, the available evidence remained insufficient to support the protective effect of fluvoxamine compared with a placebo in reducing 30% of the relative risk of clinical deterioration (Figure 6b).

**Figure 7:**
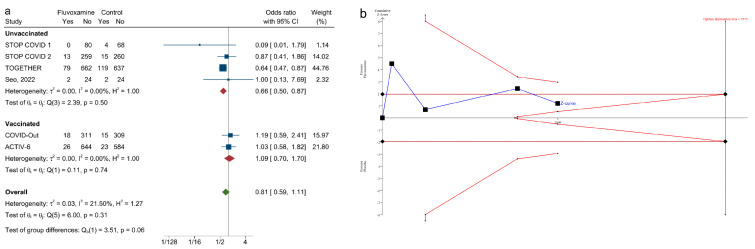
(**a**). Subgroup analysis of the unvaccinated group vs. the vaccinated group. ([6,7,8,16,17,18]) (**b**). Trial sequential analysis of the subgroup of the unvaccinated group on the primary outcome using a 30% relative risk reduction threshold.

For the subgroup analysis, the four studies [6,7,8,17] in the unvaccinated group were conducted before COVID-19 vaccine approval. On the other hand, about 52% of the participants and 67% of the participants in the COVID-Out study [16] and ACTIV-6 study [18] were vaccinated, and these two studies were classified as the vaccinated group. Fluvoxamine was associated with reduced odds of clinical deterioration in the unvaccinated group (Figure 7a; k = 4; OR: 0.66; 0.50–0.87, I2 = 0%, Q value = 2.39, *p* = 0.05) but was not in the vaccinated group (Figure 7a; k = 2, OR:1.09; 0.70–1.70, I2 = 0%). The difference between the two groups was not significant (*p* = 0.06). For the TSA in the unvaccinated group, it remains uncertain if fluvoxamine is associated with a 30% relative risk reduction in clinical deterioration when compared with the placebo (Figure 7b).

**Figure 8:**
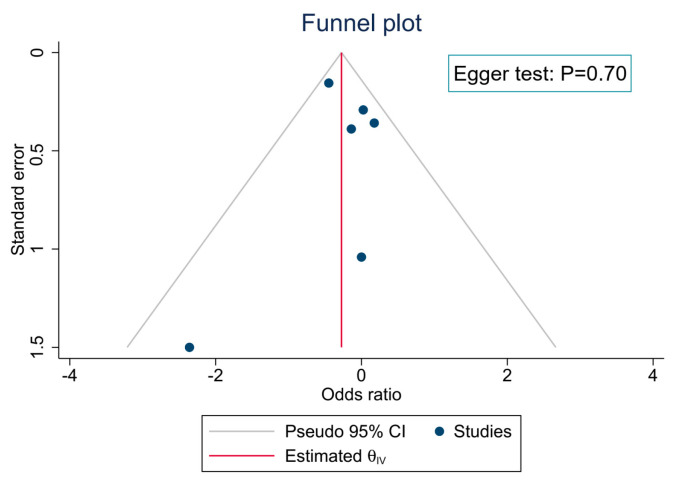
Publication bias and a small-study effect on the primary outcome.

The funnel plots and Egger’s tests were not consistent with both potential publication bias and the small-study effect for the primary outcome (Figure 8).

## 4. Discussion

In the present meta-analysis, we assessed the efficacy of fluvoxamine in reducing clinical deterioration among adults with symptomatic COVID-19 infection and used TSA to assess the reliability of such evidence. We had several findings, which are as follows: first, using a pairwise meta-analysis, the effect of fluvoxamine was not statistically significant on clinical deterioration and hospitalization. The pooled OR appeared to be driven by the TOGETHER trial, suggesting that other large-scale studies should have been conducted, and the trials conducted early in the COVID-19 pandemic might be able to detect larger effects than the more recent trials. Moreover, the extent of heterogeneity for clinical deterioration and hospitalization might be greater because the 95% CIs for the point estimate I2 are wide for both the primary and secondary outcomes. Although the two published meta-analyses suggested that fluvoxamine was associated with a reduction in the risk of clinical deterioration when compared with a placebo, [9,10] our study findings did not support this hypothesis. Second, the results of the TSA suggest that no reliable evidence indicates that fluvoxamine reduced the relative risk of clinical deterioration by 30% when compared with a placebo among adult patients with symptomatic COVID-19 infection. The reliability of a 10% or 20% relative risk reduction is also uncertain. Third, in the subgroup analysis of the unvaccinated group vs. the vaccinated group, we found that fluvoxamine was associated with reduced odds of clinical deterioration in the unvaccinated group but not in the vaccinated group. Nevertheless, the between-group difference was not significant. Fourth, when excluding the study that was unable to provide data that completely met our definition for the primary outcome, fluvoxamine was still not associated with reducing clinical deterioration in patients with symptomatic COVID-19 infection. 

In the subgroup analysis, when using a traditional meta-analysis, we found that fluvoxamine was associated with a reduction in clinical deterioration in the unvaccinated group, which was similar to the two previous meta-analyses (both included STOP COVID 1, STOP COVID 2, and TOGETHER) [6,7,8]. However, after using the TSA for adjustment type I errors in the unvaccinated group (k = 4) [6,7,8,17], the results of the TSA did not support the protective effect of fluvoxamine in reducing the relative risk of clinical deterioration by 30%. On the other hand, 52% and 67% of the participants in the two latest studies [16,18] in the vaccinated group had been vaccinated. In contrast to the unvaccinated group, fluvoxamine was not associated with a reduced risk of clinical deterioration when compared with the placebo in the vaccinated group. Probably, despite the vaccination, as time has passed, a better understanding of the virus and improvements in treatment and preventive strategies might have also played a role in lowering the risk of clinical deterioration in both the fluvoxamine and control groups of the latest two studies (unvaccinated group: fluvoxamine 8.4%, placebo 12.4%; vaccinated group: fluvoxamine 4.4%, placebo 4.1%)

When examining the effect of fluvoxamine on COVID-19, we used 30%, 20%, and 10% relative risk reduction thresholds for the TSA. These relative risk reduction thresholds were not high when compared with the effect of COVID-19 vaccines or antiviral medications. For example, in an Israel cohort study on the BNT162b2 vaccine, the results showed that 3607 participants in the unvaccinated group contracted symptomatic COVID-19, and 174 were defined as severe COVID-19 cases. In the vaccinated group, 2389 contracted symptomatic COVID-19, and 55 had severe COVID-19. The calculated relative risk reduction of severe COVID-19 in patients with symptomatic COVID-19 infection was 52.3% [31]. An RCT performed in the United States using the mRNA-1273 vaccine in 799 patients with symptomatic COVID-19 showed a 74.5% relative risk reduction of severe COVID-19 [32]. An RCT using molnupiravir in 1433 unvaccinated participants with symptomatic COVID-19 infection showed that molnupiravir reduced the relative risk of hospitalization or death by 31.0% [33]. Another RCT using nirmatrelvir plus ritonavir regarding 2085 unvaccinated participants reported eight events of hospitalization within 1039 patients and 5 days of treatment after the onset of symptoms in the intervention group, and 66 events of hospitalization in 1046 patients in the placebo group, reflecting an 87.8% relative risk reduction in hospitalization or death [34].

In the current study, we found that fluvoxamine did not reduce the relative risk of clinical deterioration by 30% among patients with symptomatic COVID-19 infection, and the effect of 10% or 20% risk reduction was still uncertain. When compared with COVID-19 vaccines or antiviral medications, the protective effect of fluvoxamine seemed to be inadequate. Notably, in the fluvoxamine trials, patients with severe symptoms of COVID had been excluded before their enrolment [7,8,16,17,18]. In contrast, such participants were included when COVID-19 infection was initially confirmed in antiviral medicine trials [33,34]. Therefore, the protective effect of fluvoxamine might be overestimated. 

There are still several ongoing trials (Table 2) identified in the registry that are testing the effect of fluvoxamine on the COVID-19 infection. Our study findings are important for these ongoing trials. Investigators and funding bodies need to consider the probable futility of conducting similar trials with a small sample size because the findings from such trials are unlikely to alter our study results. In addition, the required sample size was 11,436 participants for a 20% relative risk reduction threshold and 48,059 participants for a 10% relative risk reduction threshold. This suggests we still need several large-scale RCTs to provide reliable evidence that fluvoxamine can reduce the relative risk of clinical deterioration by 10% or 20% among patients with symptomatic COVID-19 infection. Even so, because of the small amount of relative risk reduction compared with COVID-19 vaccines and antiviral medications, prescribing fluvoxamine to treat COVID-19 infection still cannot be justified. 

### Limitations

Several limitations need to be considered. First, the protective effect of vaccination or antiviral medicine might not be comparable with fluvoxamine directly because of different study methodologies. Second, the definitions of the primary outcomes, including “hospitalization”, “emergency department visit”, or “death”, were not all applied across the studies. Notably, we could not separate the primary outcomes we defined from all of the clinical deterioration data in the STOP COVID 2 study. Third, we were still limited by a small number of RCTs, and we could not conduct other sensitivity tests, such as meta-regression.

## 5. Conclusions

Our study provides reliable evidence that fluvoxamine might not provide a 30% reduction in the relative risk of clinical deterioration among adult patients with symptomatic COVID-19 when compared with a placebo. Besides, there is still uncertainty about a 10% or 20% relative risk reduction. With the number of available COVID-19 vaccines and antiviral medications increasing, the role of fluvoxamine in treating COVID-19 needs to be reassessed.

## Figures and Tables

**Table 2:** Details of the “not included” randomized controlled trials identified in search of registry.

	Registration ID	Status	Results Available	Intervention	Region
1	NCT04718480	Recruiting	No Results Available	Drug: Placebo Drug: Fluvoxamine	Hungary
2	NCT05087381	Completed	No Results Available	Drug: Fluvoxamine Maleate 50 mg Combination: Fluvoxamine, Bromhexine Combination: Fluvoxamine, Cyproheptadine Drug: Niclosamide Combination: Niclosamide, Bromhexine	Thailand
3	NCT04885530	Recruiting	No Results Available	Drug: Ivermectin Drug: Fluvoxamine Drug: Fluticasone Other: Placebo	USA
4	IRCT20131115015405N4	Completed	No Results Available	Drug: Fluvoxamine Drug: Placebo	Iran
5	TCTR20210615002	Completed	No Results Available	Combination: Fluvoxamine with Favipiravir Drug: Favipiravir Combination: Fluvoxamine and favipiravir and dexamethasone Combination: Favipiravir and dexamethasone	Thailand

## Data Availability

The datasets analyzed during the current study are available from the corresponding author upon reasonable request.

## Supplementary Materials

The following supporting information can be downloaded at: https://www.mdpi.com/article/10.3390/ijerph20054088/s1, Supplementary S1: Preferred Reporting Items for Systematic Reviews and Meta-analyses (PRISMA) 2020 statement; Supplementary S2: Complete search strategies; Supplementary S3: Reasons for exclusion.
