# Supplementary material for: Trial Sequential Analysis and Updated Meta-Analysis of Fluvoxamine on Clinical Deterioration in Adult Patients with Symptomatic COVID-19 Infection
Source: Int J Environ Res Public Health. 2023 Feb 24;20(5):4088. doi: 10.3390/ijerph20054088 (PMC10002389; doi:10.3390/ijerph20054088)
Supplement: Supplementary file 1 [file ijerph-20-04088-s001.zip › ijerph-2079210-supplementary.pdf]

Supplementary S1. Preferred Reporting Items for Systematic Reviews and Meta-analyses (PRISMA) 2020 statement

Section and Topic	Item #	Checklist item	Location where item is reported
**TITLE**			
Title	1	Identify the report as a systematic review.	p. 1 title
**ABSTRACT**			
Abstract	2	See the PRISMA 2020 for Abstracts checklist.	p. 1 abstract
**INTRODUCTION**			
Rationale	3	Describe the rationale for the review in the context of existing knowledge.	p.2, 1st~3rd paragraph of Introduction
Objectives	4	Provide an explicit statement of the objective(s) or question(s) the review addresses.	p.2 , 4th paragraph of Introduction
**METHODS**			
Eligibility criteria	5	Specify the inclusion and exclusion criteria for the review and how studies were grouped for the syntheses.	p.3, paragraph of Search strategy and selection criteria
Information sources	6	Specify all databases, registers, websites, organisations, reference lists and other sources searched or consulted to identify studies. Specify the date when each source was last searched or consulted.	p.3, paragraph of Search strategy and selection criteria
Search strategy	7	Present the full search strategies for all databases, registers and websites, including any filters and limits used.	Appendix 2
Selection process	8	Specify the methods used to decide whether a study met the inclusion criteria of the review, including how many reviewers screened each record and each report retrieved, whether they worked independently, and if applicable, details of automation tools used in the process.	p.3, paragraph of Search strategy and selection criteria
Data collection	9	Specify the methods used to collect data from reports, including how many	p.3, paragraph of Search strategy and selection criteria

Section and Topic	Item #	Checklist item	Location where item is reported
process		reviewers collected data from each report, whether they worked independently, any processes for obtaining or confirming data from study investigators, and if applicable, details of automation tools used in the process.	
Data items	10a	List and define all outcomes for which data were sought. Specify whether all results that were compatible with each outcome domain in each study were sought (e.g. for all measures, time points, analyses), and if not, the methods used to decide which results to collect.	p.3, paragraph of Data extraction and assessment of bias
	10b	List and define all other variables for which data were sought (e.g. participant and intervention characteristics, funding sources). Describe any assumptions made about any missing or unclear information.	p.3, paragraph of Data extraction assessment of bias
Study risk of bias assessment	11	Specify the methods used to assess risk of bias in the included studies, including details of the tool(s) used, how many reviewers assessed each study and whether they worked independently, and if applicable, details of automation tools used in the process.	p.3, paragraph of Data extraction assessment of bias
Effect measures	12	Specify for each outcome the effect measure(s) (e.g. risk ratio, mean difference) used in the synthesis or presentation of results.	p.3, paragraph of Data analysis
Synthesis methods	13a	Describe the processes used to decide which studies were eligible for each synthesis (e.g. tabulating the study intervention characteristics and comparing against the planned groups for each synthesis (item #5)).	p.3, paragraph of Data analysis

Section and Topic	Item #	Checklist item	Location where item is reported
	13b	Describe any methods required to prepare the data for presentation or synthesis, such as handling of missing summary statistics, or data conversions.	p.3, paragraph of Data analysis
	13c	Describe any methods used to tabulate or visually display results of individual studies and syntheses.	p.3, paragraph of Data analysis
	13d	Describe any methods used to synthesize results and provide a rationale for the choice(s). If meta-analysis was performed, describe the model(s), method(s) to identify the presence and extent of statistical heterogeneity, and software package(s) used.	p.3, paragraph of Data analysis
	13e	Describe any methods used to explore possible causes of heterogeneity among study results (e.g. subgroup analysis, meta-regression).	p.3, paragraph of Data analysis
	13f	Describe any sensitivity analyses conducted to assess robustness of the synthesized results.	p.3, paragraph of Data analysis
Reporting bias assessment	14	Describe any methods used to assess risk of bias due to missing results in a synthesis (arising from reporting biases).	Not applicable
Certainty assessment	15	Describe any methods used to assess certainty (or confidence) in the body of evidence for an outcome.	Not applicable
**RESULTS**			
Study selection	16a	Describe the results of the search and selection process, from the number of records identified in the search to the number of studies included in the review, ideally using a flow diagram.	Figure 1 (PRISMA 2020 flow diagram)
	16b	Cite studies that might appear to meet the inclusion criteria, but which were	Appendix 2

Section and Topic	Item #	Checklist item	Location where item is reported
		excluded, and explain why they were excluded.	
Study characteristics	17	Cite each included study and present its characteristics.	Table 1
Risk of bias in studies	18	Present assessments of risk of bias for each included study.	e-Figure 1, e-Figure 2
Results of individual studies	19	For all outcomes, present, for each study: (a) summary statistics for each group (where appropriate) and (b) an effect estimate and its precision (e.g. confidence/credible interval), ideally using structured tables or plots.	Figure 2
Results of syntheses	20a	For each synthesis, briefly summarise the characteristics and risk of bias among contributing studies.	1stparagraph of Result
	20b	Present results of all statistical syntheses conducted. If meta-analysis was done, present for each the summary estimate and its precision (e.g. confidence/credible interval) and measures of statistical heterogeneity. If comparing groups, describe the direction of the effect.	Figure 2
	20c	Present results of all investigations of possible causes of heterogeneity among study results.	p.7 paragraph of Publication bias, subgroup analyses, and sesitivity test
	20d	Present results of all sensitivity analyses conducted to assess the robustness of the synthesized results.	e-Figure 3, e-Figure 5, eFigure 8-11
Reporting biases	21	Present assessments of risk of bias due to missing results (arising from reporting biases) for each synthesis assessed.	e-Figure 1, e-Figure 2
Certainty of evidence	22	Present assessments of certainty (or confidence) in the body of evidence for each outcome assessed.	e-Figure 1, e-Figure 2
**DISCUSSION**			

Section and Topic	Item #	Checklist item	Location where item is reported
Discussion	23a	Provide a general interpretation of the results in the context of other evidence.	p.7, 1st paragraph of Discussion
	23b	Discuss any limitations of the evidence included in the review.	p. 8 *Limitation*
	23c	Discuss any limitations of the review processes used.	p. 8 *Limitation*
	23d	Discuss implications of the results for practice, policy, and future research.	p. 8 paragraph of *Conclusions*
**OTHER INFORMATION**			
Registration and protocol	24a	Provide registration information for the review, including register name and registration number, or state that the review was not registered.	OSF:(10.17605/OSF.IO/ZMTS8)
	24b	Indicate where the review protocol can be accessed, or state that a protocol was not prepared.	doi: 10.17605/OSF.IO/ZMTS8
	24c	Describe and explain any amendments to information provided at registration or in the protocol.	none
Support	25	Describe sources of financial or non-financial support for the review, and the role of the funders or sponsors in the review.	none
Competing interests	26	Declare any competing interests of review authors.	none
Availability of data, code and other materials	27	Report which of the following are publicly available and where they can be found: template data collection forms; data extracted from included studies; data used for all analyses; analytic code; any other materials used in the review.	appendix

Supplementary S2. Complete search strategies

Database
**MEDLINE search strategy**
1 COVID-19/ or COVID-19.mp.
2 SARS-CoV-2/ or SARS-CoV-2.mp.
3 Severe Acute Respiratory Syndrome/ or Severe Acute Respiratory Syndrome.mp.
4 Fluvoxamine/ or Fluvoxamine.mp.
5 1 or 2 or 3
6 4 and 5 (83)
**Cochrane Central Register of Controlled Trials(CENTRAL) search strategy**
#1 MeSH descriptor: [COVID-19] explode all trees
#2 (COVID-19):ti,ab,kw
#3 MeSH descriptor: [SARS-CoV-2] explode all trees
#4 (SARS-CoV-2):ti,ab,kw
#5 MeSH descriptor: [Severe Acute Respiratory Syndrome] explode all trees
#6 (Severe Acute Respiratory Syndrome):ti,ab,kw
#7 #1 OR #2 OR #3 OR #4 OR #5 OR #6
#8 MeSH descriptor: [Fluvoxamine] explode all trees
#9 (Fluvoxamine):ti,ab,kw
#10 #8 OR #9
#11 #7 AND #10 (26)
**Embase search strategy**
#1. 'covid 19'/exp OR 'covid 19'
#2. 'sarscov 2'/exp OR 'sarscov 2'
#3. 'severe acute respiratory syndrome'/exp OR 'severe acute respiratory syndrome'
#4. #1 OR #2 OR #3
#5. 'fluvoxamine'/exp OR fluvoxamine
#6. #4 AND #5 (181)
**PsycINFO search strategy**
S1 (covid-19 or coronavirus or 2019-ncov or sars-cov-2 or cov-19) OR SARS-CoV-2 OR Severe Acute Respiratory Syndrome
S2 Fluvoxamine
S3 (Fluvoxamine) AND (S1 AND S2)(12)
**ClinicalTrials.gov (https://clinicaltrials.gov/)**
Fluvoxamine | COVID-19 (9)

MEDLINE (N=83)

[(COVID-19) or (SARS-CoV-2) or (Severe Acute Respiratory Syndrome)] And (Fluvoxamine)

Cochrane Central Register of Controlled Trials(CENTRAL) (N=26)

[(COVID-19) or (SARS-CoV-2) or (Severe Acute Respiratory Syndrome)] And (Fluvoxamine)

MeSH term: [(COVID-19) or (SARS-CoV-2) or (Severe Acute Respiratory Syndrome)] And (Fluvoxamine)

Embase search strategy (N=181)

[(COVID-19) or (SARS-CoV-2) or (Severe Acute Respiratory Syndrome)] And (Fluvoxamine)

PsycINFO search strategy (N=12)

[(covid-19) or (coronavirus) or (2019-ncov) or (sars-cov-2) or (cov-19) or (Severe Acute Respiratory Syndrome)] And (Fluvoxamine)]

ClinicalTrials.gov (https://clinicaltrials.gov/) (N=9)

(Fluvoxamine) And COVID-19

Supplementary S3. Reasons for exclusion

Comment (n=9)

1. Boulware DR, Abassi M. Fluvoxamine for the treatment of COVID-19. The Lancet Global Health. 2022;10(3):e329.
2. Dodds MG, Doyle EB, Reiersen AM, Brown F, Rayner CR. Fluvoxamine for the treatment of COVID-19. The Lancet Global Health. 2022;10(3):e332.
3. Hoertel N. Do the Selective Serotonin Reuptake Inhibitor Antidepressants Fluoxetine and Fluvoxamine Reduce Mortality among Patients with COVID-19? JAMA Network Open. 2021.
4. Kim H, Manzi S, Gonzelez-Heydrich J, Picker J. Fluvoxamine for the treatment of COVID-19. The Lancet Global Health. 2022;10(3):e330.
5. Manu P. Fluvoxamine for Acute COVID-19 Infection: Weak Hypothesis, Predictable Failure. American Journal of Therapeutics. 2022;29(3):E342-E3.
6. Marzolini C, Marra F, Boyle A, Khoo S, Back DJ. Fluvoxamine for the treatment of COVID-19. The Lancet Global Health. 2022;10(3):e331.
7. Bhimraj A, Gallagher JC. Lack of Benefit of Fluvoxamine for COVID-19. JAMA. 2023 Jan 24;329(4):291-292.
8. Bramante CT, Buse JB, Boulware DR. Trial of Metformin, Ivermectin, and Fluvoxamine for Covid-19. Reply. N Engl J Med. 2022 Dec 15;387(24):e65.
9. Shukla AK, Misra S. Trial of Metformin, Ivermectin, and Fluvoxamine for Covid-19. N Engl J Med. 2022 Dec 15;387(24):e65. doi: 10.1056/NEJMc2212542. PMID: 36516099.

Review (n=1)

10. Marčec R, Likić R. Could fluvoxamine keep COVID-19 patients out of hospitals and intensive care units? Croatian Medical Journal. 2021;62(1):95-100.

Outcome not interest (n=2)

11. Cavanna L, Citterio C. Randomised clinical trials on outpatient treatment of SARS-COV-2 infection: Light and shadows. International Journal of Clinical Practice. 2021;75(12).
12. Mills FP, Reis G, Wilson LA, Thorlund K, Forrest JI, Guo CM, Boulware DR, Mills EJ; TOGETHER Investigators. Early Treatment with Fluvoxamine among Patients with COVID-19: A Cost-Consequence Model. Am J Trop Med Hyg. 2022 Nov 15;108(1):101-106.
